# Reduced Susceptibility to Neuraminidase Inhibitors in Influenza B Isolate, Canada

**DOI:** 10.3201/eid2504.181554

**Published:** 2019-04

**Authors:** Yacine Abed, Clément Fage, Patrick Lagüe, Julie Carbonneau, Jesse Papenburg, Donald C. Vinh, Guy Boivin

**Affiliations:** Laval University, Québec City, Québec, Canada (Y. Abed, C. Fage, P. Lagüe, J. Carbonneau, G. Boivin);; Montreal Children’s Hospital, Montreal, Québec (J. Papenburg);; McGill University Health Center, Montreal (D.C. Vinh)

**Keywords:** influenza B, resistance, neuraminidase, Gly407Ser, oseltamivir, zanamivir, peramivir, viruses, Canada, influenza

## Abstract

We identified an influenza B isolate harboring a Gly407Ser neuraminidase substitution in an immunocompromised patient in Canada before antiviral therapy. This mutation mediated reduced susceptibility to oseltamivir, zanamivir, and peramivir, most likely by preventing interaction with the catalytic Arg374 residue. The potential emergence of such variants emphasizes the need for new antivirals.

Neuraminidase inhibitors (NAIs) are recommended for the control of severe influenza A and B infections (*1*). Nevertheless, antiviral resistance may emerge in immunocompromised persons, with major clinical implications (*2*).

In 2017–18, influenza B/Yamagata/16/88-like strains accounted for 50% of seasonal infections in Canada (*3*). In March 2018, we identified an influenza B/Yamagata/16/88–like variant containing a Gly407Ser NA substitution conferring reduced susceptibility to various NAIs. This variant was recovered from an immunocompromised patient before NAI therapy.

The 62-year-old woman, who had non-Hodgkin lymphoma, underwent an autologous stem cell transplant in February 2017. In March 2018, she developed therapy-related acute myeloid leukemia that failed to respond to cytarabine treatment. During hospitalization, she had influenza-like symptoms, with confirmed influenza B detection by RT-PCR. Oseltamivir (75 mg 2×/d) was administered during March 27, 2018–April 4, 2018. Because the patient’s respiratory symptoms worsened and influenza B persisted despite treatment, we replaced oseltamivir with intravenous zanamivir (600 mg 2×/d) but switched back to oseltamivir because of respiratory distress episodes. Ultimately, the patient opted to stop treatment and died a few days later.

We sequenced the viral hemagglutinin (HA) and NA genes from nasopharyngeal swab specimens using the ABI 3730 analyzer (Thermo Fisher, https://www.thermofisher.com). The HA (GenBank accession no. MH450013) and NA (GenBank accession no. MH449670) sequences from the March 27, 2018, specimen (pretherapy: B/Quebec/1182C/2018) were identical to the April 4, 2018, specimen (day 9 of oseltamivir therapy), sharing 99.5% aa identity with the HA (GenBank accession no. EPI544262) and 98.7% with the NA (GenBank accession no. EPI544263) of the B/Phuket/3073/2013 vaccine strain. Both clinical samples contained a Gly407Ser NA substitution, a marker of NAI resistance (*4*). We cloned the NA gene from pre– and post–oseltamivir therapy viruses into pJET cloning plasmid and sequenced 15 clones per virus. All NA clones contained the Gly407Ser mutation.

We used an unrelated 2018 isolate (B/Quebec/88855/2018; GenBank accession nos. MH450019 for HA, MH450017 for NA) as a wild-type control for further in vitro characterization. B/Quebec/88855/2018 (wild-type) and B/Quebec/1182C/2018 (Gly407Ser) shared 99.8% aa HA and 99.4% aa NA identities.

We determined NAI 50% inhibitory concentrations (IC_50_s) of isolates using fluorometric-based NA inhibition assays (*5*) and evaluated their NA activity (V_max_ [maximum velocity of substrate conversion]) by performing enzyme kinetics experiments (*6*). B/Quebec/1182C/2018 demonstrated reduced inhibition (RI; 5- to 50-fold increases in IC_50_ over wild-type) (*4*) to oseltamivir, zanamivir, and peramivir, showing 5.97-, 32.44-, and 38.34-fold increases in IC_50_s, respectively, over B/Quebec/88855/2018 WT (Table). The last 2 isolates had similar NA activity (V_max_) (Table). To confirm the role of the Gly407Ser mutation, we expressed the recombinant wild-type and Gly407Ser mutant proteins (obtained by PCR-mediated mutagenesis) in 293T cells (*7*) and found that Gly407Ser also increased oseltamivir, zanamivir, and peramivir IC_50 _levels by 4.16-, 10.07- and 16.36-fold, respectively (Table).

**Table:** Susceptibility profiles and NA activity of influenza B virus isolates and susceptibility profiles of recombinant influenza B NAs determined by assays using the fluorescent MUNANA substrate, Canada*

Sample type	IC_50_ in nM + SD (fold increase) [phenotype]†			NA activity, V_max_‡
	Oseltamivir	Zanamivir	Peramivir	
Clinical isolate				
B/Phuket/3073/2013, vaccine	18.98 + 3.89	0.70 + 0.17	0.74 + 0.02	ND
B/Québec/88855/2018, WT	17.47 ± 1.43 (1) [NI]	0.85 ± 0.09 (1) [NI]	0.92 ± 0.09 (1) [NI]	2.24 ± 0.3
B/Québec/1182C/2018, Gly407Ser	104 + 14.62 (5.97) [RI]	27.58 + 2.56 (32.44) [RI]	26.08 + 0.1 (38.34) [RI]	2.18 + 0.47
Recombinant neuraminidase				
B/Quebec/88855/2018, WT	11.16 + 5.25 (1) [NI]	0.97 + 0.27 (1) [NI]	0.76 + 0.19 (1) [NI]	ND
B/Quebec/88855/2018, Gly407Ser	46.52 + 12.58 (4.16) [NI]	9.77 + 0.90 (10.07) [RI]	12.44 + 5.47 (16.36) [RI]	ND

*Values are from a representative experiment performed in duplicate. IC50, 50% inhibitory concentration; MUNANA, 2 '-(4-methylumbelliferyl)-α-D-N-acteylneuraminic acid; NA, neuraminidase; NAI, neuraminidase inhibitor; ND, not done; NI, normal inhibition (<5-fold increase in IC50 over WT); RI, reduced inhibition (5- to 50-fold increase in IC50 over WT); Vmax, maximum velocity of substrate conversion; WT, wild type. †The phenotype of susceptibility to NAI following the World Health Organization guidelines.  ‡Numbers indicate mean Vmax values (U/sec) ± SD of a kinetics experiment performed in triplicate.

We next evaluated replication kinetics of the wild-type and Gly407Ser isolates in ST6GalI-MDCK cells. Mean viral titers obtained with wild-type isolates were higher than the mutant at 24 and 48 h postinfection (p<0.01); comparable titers were obtained at 72 and 96 h postinfection (Appendix Figure 1). To assess genetic stability, we sequenced the HA/NA genes after 4 passages in ST6GalI-MDCK cells and found that Gly407Ser was conserved with no additional sequence alterations, suggesting genetic stability of the NA mutant. 

Finally, we performed molecular dynamics simulations for deciphering the mechanism of cross-RI displayed by Gly407Ser (Appendix). Our model suggests that Gly407Ser affects interaction networks involving a key arginine residue within the NA active site (Arg374) (*8*) and neighboring residues (Appendix Figure 2). In the wild-type protein, Arg374 forms hydrogen bonds with NAIs (Appendix Figure 2, panels A–C). There is a hydrogen bond between the Gly407 amine and the Arg374 carbonyl, and between the Trp408 amine and the Glu428 carbonyl, in addition to hydrophobic interactions between Trp408 and Val430 side chains. In the Gly407Ser variant, the orientation of Arg374 prevents hydrogen bond formation with NAIs (Appendix Figure 2, panels D–F). A hydrogen bond exists between the Ser407 amine and the Arg374 carbonyl and between the Ser407 side chain hydroxyl and the Glu428 carbonyl, in addition to hydrophobic interactions between the Trp408 and the Arg374 side chains.

In 2007, a Gly407Ser influenza B variant was recovered from a child after 3 days of oseltamivir therapy (*9*). That variant displayed 4- and 7-fold increases in oseltamivir and zanamivir IC_50 _levels, respectively, with an unexplained mechanism (*9*). Here, we identified a contemporary Gly407Ser influenza B variant in a patient before NAI therapy and propose a molecular mechanism for such a cross-RI phenotype. We cannot exclude nosocomial transmission of this virus despite evidence for some alteration in replication kinetics. The Gly407Ser mutation was detected in the absence of NAI constituting 100% of sequenced clones, did not affect NA activity, and was conserved after in vitro passages. Thus, such a variant may retain efficient transmissibility. Nevertheless, the effect of this mutation in a suitable animal model (ferrets) remains to be assessed. The potential for emergence of variants with cross-RI to available NAIs in the absence of treatment emphasizes the need for novel antiviral strategies (including combinations) against influenza B viruses.

Appendix Additional information on reduced susceptibility to neuraminidase inhibitors in influenza B isolate, Canada.
