# Supplementary material for: Reduced Susceptibility to Neuraminidase Inhibitors in Influenza B Isolate, Canada
Source: Emerg Infect Dis. 2019 Apr;25(4):838–40. doi: 10.3201/eid2504.181554 (PMC6433030; doi:10.3201/eid2504.181554)
Supplement: Appendix — Additional information on reduced susceptibility to neuraminidase inhibitors in influenza B isolate, Canada. [file 18-1554-Techapp-s1.pdf]

Article DOI: https://doi.org/10.3201/eid2504.181554

Reduced Susceptibility to Neuraminidase Inhibitors in Influenza B Isolate, Canada

Appendix

Molecular Dynamics Simulations

Molecular dynamics simulations were performed for highlighting the mechanism of cross resistance displayed by the Gly407Ser mutation. A National Center for Biotechnology Information Protein Data Bank (NCBI PDB) BLAST (https://blast.ncbi.nlm.nih.gov/Blast.cgi) of the B/Quebec/1182C/2018 NA amino acid sequence identified B/Brisbane/60/2008 structure PDB IDs having the best sequence identity (94.8%), including 4CPL (uncomplexed NA), 4CPM (NA-oseltamivir), and 4CPN (NA-zanamivir). The amino acid sequence alignment was used to build a SWISS-MODEL (https://swissmodel.expasy.org/). Then, the WT and Gly407Ser systems were built using CHARMM-GUI (*1*) as previously described. Simulations were performed with the NAMD 2.12b1 (*2*) software using the CHARMM36m force field, TIP3P waters, a time step of 2 femtoseconds, and periodic boundary conditions. Nonbonded pair lists were updated at every step, and coordinates were saved every 2 picoseconds (ps) for analysis. Cutoffs for the short-range electrostatics and the Lennard-Jones interactions were 12 Å, with the latter smoothed via a switching function over the range of 10–12 Å. Long-range electrostatics were calculated via the particle mesh Ewald (PME) method, using a sixth-order interpolation and a grid spacing of ≈1 Å. Langevin damping with a coefficient of 1 ps–1 was used to maintain a constant temperature of 37°C, and the pressure was controlled by a Nosé-Hoover Langevin piston at 1 atm. The length of the bonds between hydrogens and heavy atoms were constrained using SETTLE for water molecules, and SHAKE for all other molecules. For each system, 3 trajectories of 150 nanoseconds (ns) were recorded, and the last 50 ns of each trajectory was used for analysis.

References

1. Jo S, Kim T, Iyer VG, Im W. CHARMM-GUI: a web-based graphical user interface for CHARMM. J Comput Chem. 2008;29:1859–65. http://dx.doi.org/10.1002/jcc.20945

2. Phillips JC, Braun R, Wang W, Gumbart J, Tajkhorshid E, Villa E, et al. Scalable molecular dynamics with NAMD. J Comput Chem. 2005;26:1781–802. http://dx.doi.org/10.1002/jcc.20289

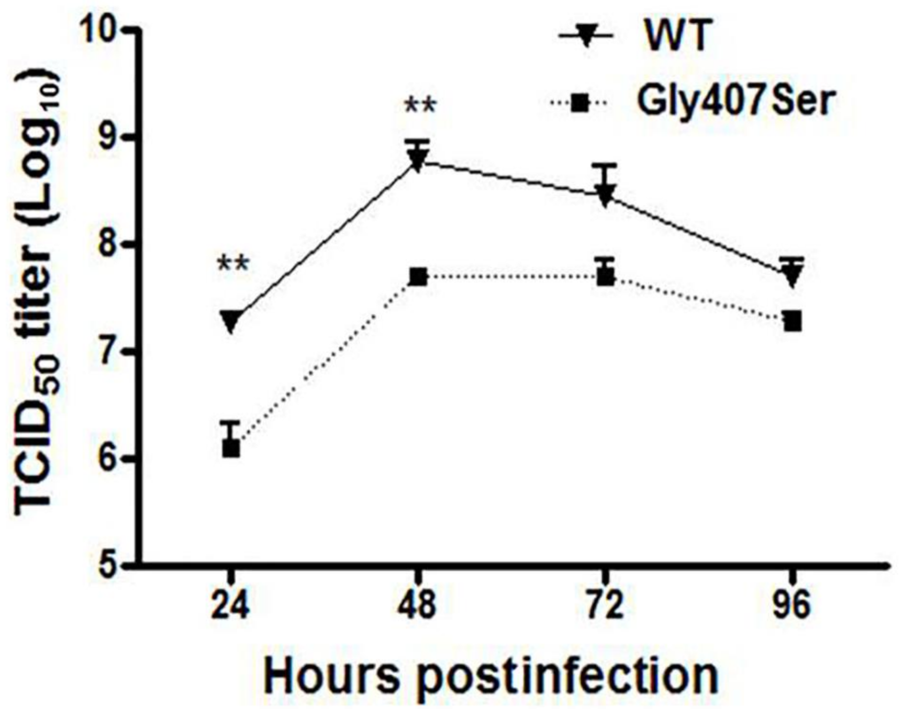

Appendix Figure 1. Replicative properties of influenza B isolates in vitro. Confluent ST6GalI-MDCK cells were infected with the WT and Gly407Ser influenza B isolates at a multiplicity of infection (MOI) of 0.001 PFU/cell. Supernatants were harvested at the indicated times and titrated by $TCID_{50}$ assays. Mean viral titers of triplicate $\pm$SD are shown. ** $p < 0.01$.

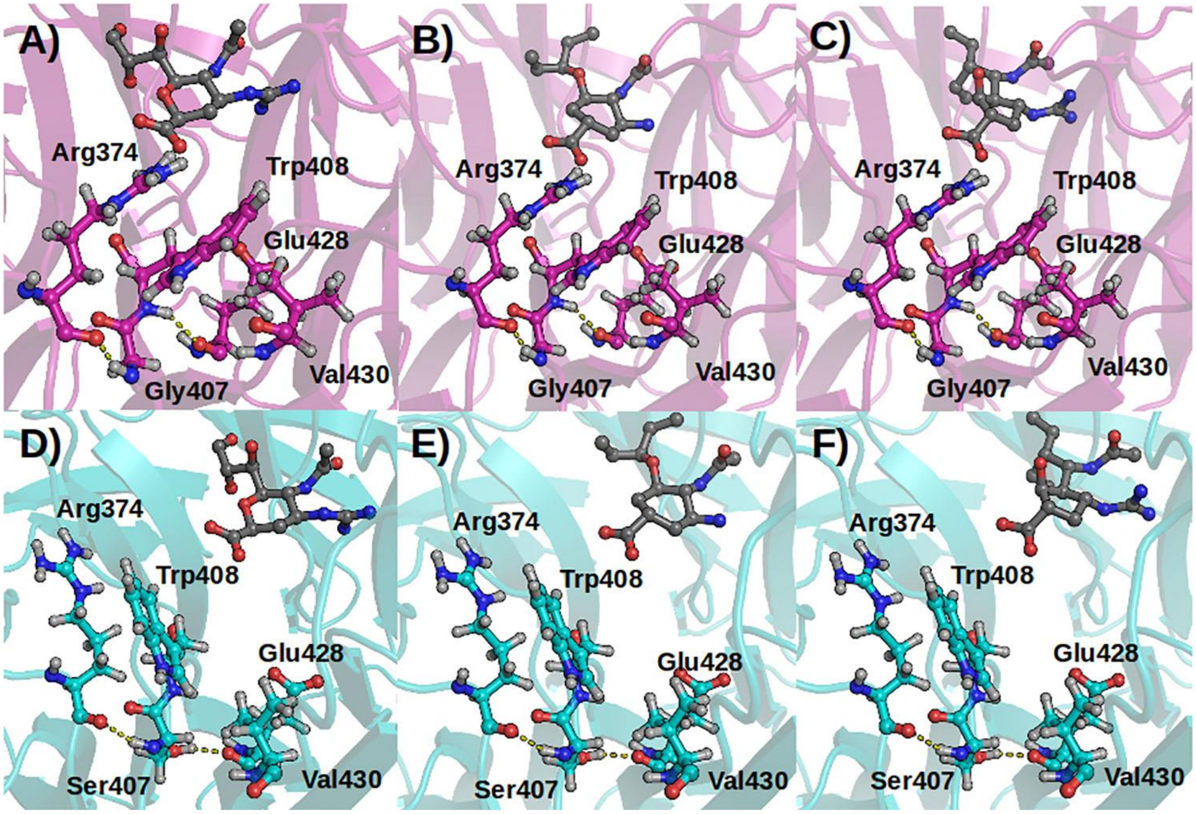

Appendix Figure 2. Trajectory analysis and molecular dynamics simulations of the Gly407Ser variant. Typical structures of the WT (in pink) and the Gly407Ser variant (in cyan). The residues involved in the structural changes from the Gly407Ser mutations are in sticks and spheres. The molecules of zanamivir (A and D), oseltamivir (B and E), and peramivir (C and F) were included to illustrate the potential interactions with Arg374. The molecules, represented with gray sticks and spheres, were positioned according to a structural alignment of the PDB 4CPN (PMID 24795482) to both the WT and the Gly407Ser variant. For the Gly407Ser variant, Arg374 is locked in an orientation that prevents hydrogen bond formation with NAIs.
